# Perspective: voltage control of magnetization in multiferroic heterostructures

**DOI:** 10.1093/nsr/nwz047

**Published:** 2019-04-01

**Authors:** Jia-Mian Hu, Ce-Wen Nan, Long-Qing Chen

**Affiliations:** 1 Department of Materials Science and Engineering, University of Wisconsin–Madison, USA; 2 State Key Lab of New Ceramics and Fine Processing, School of Materials Science and Engineering, Tsinghua University, China; 3 Department of Materials Science and Engineering, Pennsylvania State University, USA

Multiferroic materials host ferromagnetic and ferroelectric orders either in the same phase, or separately in distinct magnetic and ferroelectric phases. In some multiferroic materials, the two distinct ferroic orders can be strongly coupled (namely, magnetoelectric coupling) [1], which can be exploited for achieving new functionalities. For example, it allows for controlling magnetization with a voltage rather than a magnetic-field-generating electric current, providing an energy-efficient route to writing data in a magnetic memory [2]. This perspective focuses on such voltage control of magnetization in multiferroic heterostructures (see their different architectures in Fig. 1a–d). It briefly discusses its mechanisms, current trends, and future directions. Other topics of multiferroics are available in recent reviews [1,3–5] or other articles in this special issue. Compared to single-phase multiferroics, multiferroic heterostructures typically display a larger magnetization that is more efficient to control by voltage at room temperature (i.e. having a larger figure of merit ${\alpha _{ij}}$, defined in the next section).

**Figure 1.**
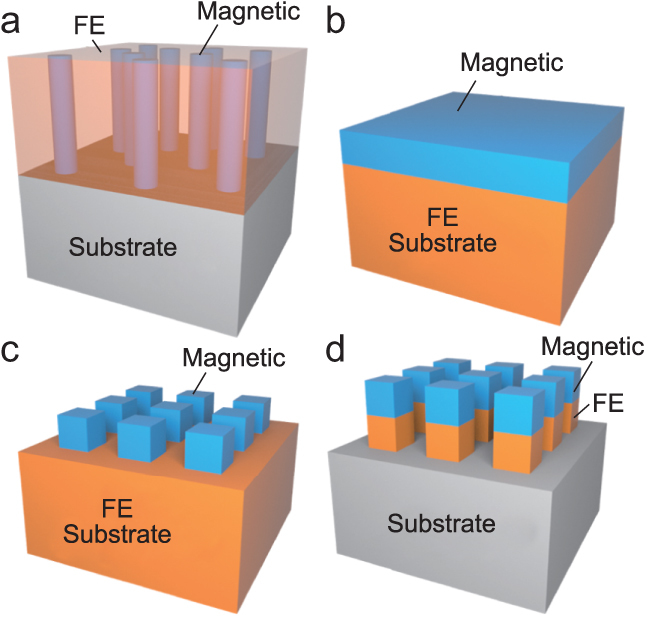
Schematics, not to scale, of (a, b) continuous multiferroic heterostructures and (c, d) patterned multiferroic nano-heterostructures for realizing voltage control of magnetization. FE: ferroelectric.

## MECHANISMS

The figure of merit for the voltage control of magnetization (*M*) is given as ${\alpha _{ij}} = {\mu _0}d{M_i}/d{E_j}$, where ${\mu _0}$ is the vacuum permeability and ${E_j}$ is the electric field established along a given direction (e.g. $j$ = *x*, *y*, *z*) as a voltage is applied. Thus, the voltage control of magnetization indicates an electric-to-magnetic energy conversion. In multiferroic ferromagnet/ferroelectric bilayer heterostructures, the mechanisms of such energy conversion can be summarized by rewriting ${\alpha _{ij}}$:

(1) \begin{eqnarray*} &&{\mu _0}d\ {M_i} = \Bigg[ \left( {\frac{{\partial {M_i}}}{{\partial \sigma _{jk}^{{\rm{FM}}}}}} \right){\eta _{\rm{\sigma }}}\left( {\frac{{\partial \sigma _{jk}^{{\rm{FE}}}}}{{\partial {E_j}}}} \right) \nonumber\\ && \quad +\, \left( {\frac{{\partial {M_i}}}{{\partial {q_{\rm{s}}}}}} \right){\eta _{\rm{q}}}\left( {\frac{{\partial {q_{\rm{c}}}}}{{\partial {E_j}}}} \right) \nonumber\\ && \quad +\, \left( {\frac{{\partial {M_i}}}{{\partial H_j^{{\rm{EC}}}}}} \right)\left( {\frac{{\partial H_j^{{\rm{EC}}}}}{{\partial {E_j}}}} \right) \nonumber\\ && \quad +\, \left( {\frac{{\partial {M_i}}}{{\partial \xi }}} \right)\left( {\frac{{\partial \xi }}{{\partial {E_j}}}} \right) \Bigg]\ d{E_j}. \end{eqnarray*}

**Figure 2.**
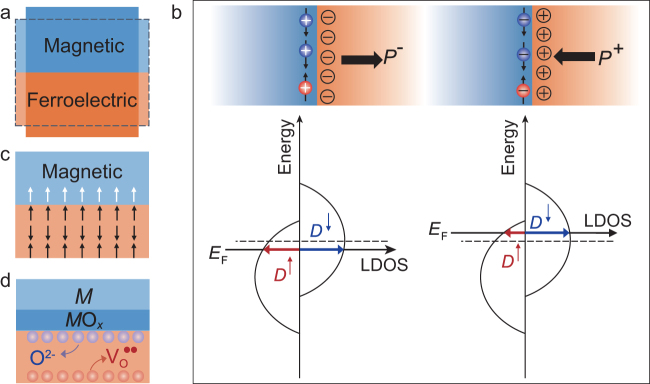
(a) Stress (strain)-mediated mechanism, which is a long-range effect and not limited to the magnet/ferroelectric interface. This is different from the other mechanisms shown herein. (b) Charge-mediated mechanism. Switching the polarization modifies the charge density of the magnetic phase near the interface via Coulomb interaction (see figures on the top), and hence tunes the interfacial spin polarization by shifting the Fermi level *E*_F_ (see figures on the bottom). (c) Exchange-coupling-mediated mechanism. Only one single antiferromagnetic (AFM) domain with perpendicular sublattice magnetization (see arrows in the bottom layer) is shown for simplicity. Electrical switching of the AFM domain can reverse the uncompensated magnetization (see arrows in the first row) on the surface of the AFM domain, further reorienting the magnetization (white arrows) in the exchange-coupled ferromagnetic domain. Details of electric-field control of AFM orders vary in different ferroelectric antiferromagnets (including YMnO_3_, LuMnO_3_ and BiFeO_3_). (d) Mechanism based on voltage control of interface chemistry, e.g. an interfacial redox reaction involving 3D transitional metals *M* or their alloys (e.g. *M* = Co, Fe, CoFe). (a, c, d were modified from [6]; b was modified with permission from [4]).

The first term in Eq. (1) suggests that the electric-to-magnetic energy conversion can occur through the transmission of an electric-field-induced elastic stress from the ferroelectric phase ($\sigma _{jk}^{{\rm{FE}}}$) to the magnetic phase (Fig. 2a); ${\eta _{\rm{\sigma }}}$ is the stress transmission efficiency ($\sigma _{jk}^{{\rm{FM}}} = {\eta _{\rm{\sigma }}}\ \sigma _{jk}^{{\rm{FE}}}$) across the interface, which depends on the size, geometry, and bulk mechanical properties of both phases, the mechanical properties of their interface, as well as the geometry and mechanical boundary conditions of the heterostructure. $( {\partial {M_i}/\partial \sigma _{jk}^{{\rm{FM}}}} )$ describes the magnetoelastic coupling of the magnetic phase, while $( {\partial \sigma _{jk}^{{\rm{FE}}}/\partial {E_j}} )$ describes the electromechanical coupling of the ferroelectric phase. To ensure an efficient stress (strain) transfer, the ferroelectric typically needs to be elastically stiff (i.e. having a relatively large elastic stiffness coefficient). For example, a ferroelectric soft polymer may generate a relatively large strain via converse piezoelectric or electrostrictive effect, but the stress $\sigma _{jk}^{{\rm{FE}}}$ that it generates would be small and hence may not induce appreciable change in the magnetization of its adjacent magnetic layer.

The second term in Eq. (1) suggests an energy conversion via the modulation of interfacial spin-polarized charge densities of the magnetic phase (${q_{\rm{s}}}$) when ${E_j}$ modulates the interfacial polarization charge densities of the ferroelectric phase (${q_{\rm{c}}}$). $( {\partial {q_{\rm{c}}}/\partial {E_j}} )$ is the interfacial electric susceptibility of the ferroelectric phase. The conversion efficiency of these two charge densities ${\eta _{\rm{q}}} = {q_{\rm{s}}}/{q_{\rm{c}}}\ $ (0 ≤ ${\eta _{\rm{q}}}$ ≤ 1) is electric-field tunable, and can be approximated as the interfacial spin polarization of the magnetic phase ${\eta _{\rm{s}}}$ as discussed below. As illustrated in Fig. 2b, the presence of a polarization enhances (top left) or reduces (top right) the interfacial electron densities in the ferroelectric phase (${q_{\rm{c}}}$), which reduces ($\Delta q_{\rm{c}}^{\rm{m}} < 0$) or enhances ($\Delta q_{\rm{c}}^{\rm{m}} > 0$) the interfacial electron densities in the magnetic phase ($q_{\rm{c}}^{\rm{m}}$) due to Coulomb interaction. The associated shift in Fermi energy in the magnetic phase can be estimated as $\Delta {E_{\rm{F}}} \approx {k_{\rm{B}}}Tln[ {( {{q_0} + \Delta q_{\rm{c}}^{\rm{m}}} )/{q_0}} ]$ [6], where ${k_{\rm{B}}}$ is the Boltzmann constant and ${q_0}$ the interfacial electron density in the magnetic phase in the absence of polarization. Thus, $\Delta {E_{\rm{F}}} < 0$ for $\Delta q_{\rm{c}}^{\rm{m}} < 0$ (bottom left), while $\Delta {E_{\rm{F}}} > 0$ for $\Delta q_{\rm{c}}^{\rm{m}} > 0$ (bottom right), where the dashed line indicates the ${E_{\rm{F}}}$ in the absence of polarization. A shifted ${E_{\rm{F}}}$ would alter the interfacial spin polarization $\ {\eta _{\rm{s}}} = ( {{D^ \downarrow } - {D^ \uparrow }} )/( {{D^ \downarrow } + {D^ \uparrow }} )$, where ${D^ \downarrow }$ and ${D^ \uparrow }$ are the local density of states (LDOS) of the majority spin-down and minority spin-up electrons. The associated change in the spin-polarized charge densities, $\Delta {q_{\rm{s}}}$, may be estimated as $\Delta {q_{\rm{s}}} \approx \Delta q_{\rm{c}}^{\rm{m}}{\eta _{\rm{s}}} = - \Delta {q_{\rm{c}}}{\eta _{\rm{s}}}$. Finally, $( {\partial {M_i}/\partial {q_{\rm{s}}}} )$ describes the magnetization change induced by the changes in ${q_{\rm{s}}}$, which can occur via different microscopic mechanisms for different magnetic/ferroelectric interfaces (detailed discussion available in [6]).

Note that the mechanism of voltage control of magnetization illustrated in Fig. 2b is also applicable to a ferromagnet/dielectric (e.g. MgO) heterostructure. A tabular summary of relevant experimental work is given in [6]. Consider a ferromagnet/MgO heterostructure as an example. First, in contrast with a ferromagnet/ferroelectric heterostructure, charge-mediated voltage control of magnetization in the ferromagnet/MgO heterostructure is normally volatile. This is because MgO, like other non-ferroelectric dielectrics, displays a zero remanent electrical polarization. Thus the electric-field-induced changes in ${q_{\rm{c}}}$ are volatile. A charge-mediated voltage-driven 180° magnetization switching is, however, non-volatile due to the energy barrier between two magnetization vectors of different polarity. Achieving such 180° magnetization switching typically requires using a sub-nanosecond pulse voltage of precisely controlled timing (e.g. [7]). A simultaneous application of a bias magnetic field is also required, which may cause cross-talk between neighboring units in miniaturized devices. Second, the $( {\partial {q_{\rm{c}}}/\partial {E_j}} )$ of MgO is typically much smaller than those of perovskite ferroelectrics like BaTiO_3_. Therefore, to achieve the same amount of change in the interfacial charge densities $\Delta {q_{\rm{c}}}$, a higher driving electric field is needed in ferromagnet/MgO heterostructures. This may increase the risk of dielectric breakdown. However, the size scalability of ferromagnet/MgO heterostructures is much better than that of ferromagnet/BaTiO_3_ (or other complex ferroelectric oxide-based) heterostructures.

The third term in Eq. (1) describes an energy conversion that occurs when (i) the ferroelectric phase is also antiferromagnetic (AFM) and (ii) an electric field can switch the AFM order and thereby the interfacial exchange coupling field ($H_j^{{\rm{EC}}}$) between the AFM order and the ferromagnetic order (Fig. 2c), described by $( {\partial H_j^{{\rm{EC}}}/\partial {E_j}} )$. $( {\partial {M_i}/\partial H_j^{{\rm{EC}}}} )$ is the interfacial magnetic susceptibility of the magnetic phase. Note that the mechanism shown in Fig. 2c is also applicable to a ferromagnet/Cr_2_O_3_ heterostructure (see a recent experiment with Cr_2_O_3_ thin films in [8]). Such ferromagnet/Cr_2_O_3_ heterostructure is, however, not multiferroic, because Cr_2_O_3_ is magnetoelectric but not ferroelectric. Moreover, since the free energy change associated with the switching of a single AFM domain ($\Delta F$) is proportional to ${E_i}{H_i}$ in a Cr_2_O_3_ single crystal [8], simultaneous application of a bias magnetic field is needed to lift the symmetry of $\Delta F$, such that the AFM domain can be switched. Likewise, the use of a bias magnetic field would hamper device miniaturization.

Lastly, if the ferroelectric phase has a relatively high concentration of ionic defects (e.g. oxygen vacancies), the electric-field-induced transport of these ionic defects to and from the interface may enable a voltage-controllable interface chemistry, e.g. an interfacial redox reaction (Fig. 2d, described by $\partial \xi /\partial {E_j}$, where $\xi $ is the extent of the reaction). The influence of such an interfacial chemical reaction on the magnetization of the magnetic phase is described by $\partial {M_i}/\partial \xi $, whose microscopic mechanisms remain unclear [6].

Which mechanism(s) of these four dominate the voltage control of magnetization in a multiferroic heterostructure depends on specific materials systems, sizes, and operating conditions [4,6]. For instance, the charge-mediated, exchange-coupling-mediated, and interface-chemistry-mediated mechanisms are all interfacial effects, which would yield an appreciable ${\alpha _{ij}}$ only when the magnetic phase is relatively thin. In addition, the stress (strain)-mediated and the charge-mediated mechanisms (the first two terms in Eq. (1)) necessarily exist in any multiferroic heterostructures because all ferroelectrics are piezoelectrics.

## TRENDING: VOLTAGE CONTROL OF MAGNETIZATION AT THE NANOSCALE

Scaling down the size of a magnet in voltage-controlled spintronic devices can reduce energy consumption and increase packing density. This is the technological driver behind the shifting research focus from continuous heterostructures (Fig. 1a and b) to patterned nano-heterostructures (Fig. 1c and d) over the past few years.

This trend is manifested by recent computational and experimental studies, and has been pointed out in a recent *MRS Bulletin* issue [9]. Yet much remains to be done. For example, voltage-driven 180° magnetization switching with no magnetic field has been experimentally demonstrated in CoFe/BiFeO_3_ thin-film heterostructures (discussed in [5]), but not yet in patterned multiferroic nano-heterostructures. Computationally, it has been predicted that such magnetic-field-free voltage-driven 180° magnetization switching can be achieved through different routes (mechanisms) in patterned multiferroic nano-heterostructures composed of various materials systems [6], but experimental demonstrations have remained elusive.

Moreover, there are many remaining questions on the dynamics of voltage-driven magnetization switching in patterned nano-heterostructures. For example, what is the highest possible speed that one can achieve? How would such a speed limit be different for the different mechanisms of voltage-driven magnetization switching mentioned above? How can the dynamics of ferroic (ferromagnetic, ferroelectric, ferroelastic) domain walls (if any) be predicted and characterized, and how does such wall dynamics influence the overall switching speed? Can we precisely control the switching speed through a rational design of individual materials, heterostructures, and the operating conditions? Consider strain-mediated voltage-driven magnetization switching as an example. A 3D finite-element model that fully couples the elastodynamics and magnetization dynamics has been developed to predict the speed of piezostrain-mediated voltage-driven magnetization switching [9]. However, there is still a lack of computational models that fully couple elastodynamics, magnetization dynamics, and polarization dynamics. Such a model would be necessary if voltage-driven polarization switching occurs in ferroelectrics. Experimentally, it is important to characterize the dynamics of voltage-driven magnetization switching through appropriate *in situ* and time-resolved methods (e.g. electrical transport measurement, X-rays, spectroscopy, microscopy, laser-based probes). It would be particularly desirable to simultaneously image the voltage-controlled evolution of both ferroelectric and magnetic domains with high temporal and spatial resolution in patterned nano-heterostructures, such that the synergy between computational modeling and characterization can be established.

Furthermore, there are many challenges for realizing practical device applications based on voltage-driven magnetization switching in multiferroic nano-heterostructures (a few key ones have been outlined in [5]). We will discuss these challenges in detail in a separate paper. Overall, fueled by recent advancements in computational modeling across spatiotemporal scales, fabrication of high-quality magnetic and ferroelectric nanostructures, and non-destructive imaging of both the ferroelectric and magnetic domains (e.g. [10–12]), new breakthroughs in voltage control of magnetization at the nanoscale are on the horizon.

## SOME FUTURE DIRECTIONS

Research into voltage control of magnetization in multiferroic heterostructures is expected to grow steadily in the next 5 to 10 years, driven by the quest for energy-efficient spintronic devices that can be used not just for data storage but also new computing architectures (e.g. neuromorphic computing). Three possible directions are outlined below.

- *Voltage control of antiferromagnetism*, intertwined with the rapidly growing field of antiferromagnetic spintronics, can potentially lead to the development of THz energy-efficient spintronic devices. This is because the frequency of antiferromagnetic resonance is in the THz range (cf. GHz for ferromagnetic resonance). A method towards realizing strain-mediated voltage-driven switching of antiferromagnetic orders in antiferromagnetic/ ferroelectric heterostructures has recently been proposed [13].
- *Voltage control of magnetic skyrmions*, intertwined with the surging research efforts on magnetic skyrmions, can enable energy-efficient skyrmion-based electronic devices. Nationwide research efforts on magnetic skyrmions have recently been launched in the UK (the Skyrmion Project) and Germany (the Skyrmionics Priority Programme), along with substantial research expenditures in many other countries. Recently, strain-mediated voltage control of magnetic skyrmions in multiferroic nano-heterostructures (Fig. 1c and d) has been computationally demonstrated [14].
- *Voltage control of 2D magnetism*, intertwined with the surging research efforts on magnetism in 2D van der Waals materials, may also lead to new fundamental science and device concepts. Recently, voltage-controlled switching between antiferromagnetic and ferromagnetic states has been experimentally demonstrated in 2D CrI_3_ [15]. Research into the voltage control of magnetism in 2D-magnet/ferroelectric multiferroic heterostructures can therefore be anticipated.
